# Picky eating in an obesity intervention for preschool-aged children – what role does it play, and does the measurement instrument matter?

**DOI:** 10.1186/s12966-019-0845-y

**Published:** 2019-09-03

**Authors:** Pernilla Sandvik, Anna Ek, Karin Eli, Maria Somaraki, Matteo Bottai, Paulina Nowicka

**Affiliations:** 1 Department of Food Studies, Nutrition and Dietetics, Uppsala University, Box 560, 751 22 Uppsala, Sweden; 2 Division of Pediatrics, Department of Clinical Science, Intervention and Technology (CLINTEC), Karolinska Institutet, Stockholm, Sweden; 3 Social Science and Systems in Health, Division of Health Sciences, Warwick Medical School, University of Warwick, Warwick, England; 4 Unit for Biocultural Variation and Obesity, School of Anthropology and Museum Ethnography, University of Oxford, Oxford, England; 5 Division of Biostatistics, Institute of Environmental Medicine, Karolinska Institutet, Stockholm, Sweden

**Keywords:** Child eating behavior questionnaire, Food fussiness, Lifestyle behavior checklist, Parents, Randomized controlled trial

## Abstract

**Introduction:**

Research on picky eating in childhood obesity treatment is limited and inconsistent, with various instruments and questions used. This study examines the role of picky eating in a randomized controlled obesity intervention for preschoolers using subscales from two instruments: The Child Eating Behavior Questionnaire (CEBQ) and the Lifestyle Behavior Checklist (LBC).

**Method:**

The study includes 130 children (mean age 5.2 years (SD 0.7), 54% girls, mean Body Mass Index (BMI) z-score 2.9 (SD 0.6)) and their parents (nearly 60% of non-Swedish background, 40% with university degree). Families were randomized to a parent-group treatment focusing on evidence-based parenting practices or to standard treatment focusing on lifestyle changes. The children’s heights and weights (BMI z-score) were measured at baseline, and at 3, 6 and 12 months post baseline. At these time-points, picky eating was reported by parents using the CEBQ (Food Fussiness scale, 6 items) and 5 items from the LBC. Child food intake was reported with a Food Frequency Questionnaire (FFQ). Pearson correlation was used to study associations between baseline picky eating and baseline BMI z-scores and food intake. Mixed effects models were used to study associations between the two measurements of picky eating and changes in picky eating, to assess the effects of changes in picky eating on BMI z-scores, and to evaluate baseline picky eating as a predictor of changes in BMI z-scores.

**Results:**

Neither the standard treatment nor the parent-group treatment reduced the degree of picky eating (measured with CEBQ or LBC). Baseline picky eating measured with the CEBQ was associated with a lower BMI z-score and lower intake of vegetables. Children with a higher degree of picky eating at baseline (measured with the CEBQ) displayed a lower degree of weight loss. When degree of picky eating was examined, for 25% of the children, the CEBQ and the LBC yielded diverging results.

**Conclusions:**

Baseline picky eating may weaken the effectiveness of obesity treatment, and assessments should be conducted before treatment to adjust the treatment approach. Different measurements of picky eating may lead to different results. The CEBQ seems more robust than the LBC in measuring picky eating.

**Trial registration:**

Clinicaltrials.gov, NCT01792531. Registered 15 February 2013 - Retrospectively registered, https://clinicaltrials.gov/ct2/show/NCT01792531

## Background

Picky/fussy or choosy eating refers to a child’s unwillingness to eat familiar foods or try new foods, with negative impacts on children and parents in their daily activities [1]. Research on the roles of picky eating in childhood obesity is limited. A review has suggested that if disliked food is replaced by more palatable, less healthy (high in fat or sugar) alternatives, picky eating could increase the risk of overweight and obesity [2]. However, a systematic review of the effects of picky eating on weight has shown mixed results, including positive, negative and no associations [3]. According to a recent literature review, most studies have found that the prevalence of picky eating is lower among children with overweight and obesity [2]; however, two cross-sectional studies have found higher levels of picky eating among children with overweight and obesity [4, 5]. We have recently published data from Sweden showing a significantly lower prevalence of picky eating in preschoolers with obesity compared to children with underweight, normal weight and overweight [6]. Still, one third of the children with obesity in our study met the criteria for moderate picky eating, using the cut-off in the Food Fussiness (FF) subscale of the Child Eating Behavior Questionnaire (CEBQ) proposed by Steinsbekk et al. [7]. Although the prevalence of picky eating might be lower among children with obesity, this behavior could play an important role for children experiencing the double burden of picky eating and obesity. While picky eaters have a lower degree of obesogenic eating behaviors, they are more likely to complain about being physically active and to have more problematic screen time behaviors compared to children with obesity who are not picky eaters [6]. Of note, parents of children with obesity who are classified as picky eaters are more likely to perceive their child as having normal weight compared to children with obesity classified as non-picky eaters [6]. This indicates that parents may perceive picky eating as more problematic than their child’s weight [6].

Only one previous study has focused on the relationship between picky eating and the outcomes of treatment for childhood obesity. Hayes et al. [8] showed that a reduction in the degree of picky eating directly after a family-based obesity treatment was associated with a greater reduction in weight status in 7- to 11-year-old children. While Hayes et al.’s [8] family-based obesity treatment did not target picky eating specifically, based on the results, they suggest that addressing picky eating may be important in childhood obesity treatment. The findings need to be confirmed in other studies. Given that obesity interventions have been shown to be most effective in younger ages [9] and that picky eating is more commonly seen in younger children [10], it is important to explore the role of picky eating in obesity interventions for preschoolers.

A recent study has highlighted the role of a bi-directional relationship between parents and children in relation to child eating behaviors, including picky eating [11]. The study’s authors concluded that health professionals should provide parents with alternative strategies and feeding practices to cope with the emergence of neophobic and potentially picky eating behavior [11]. In the randomized controlled trial (RCT) on which the present study is based, the effects of a parent-group treatment for childhood obesity, focused on evidence-based parenting practices, was compared to standard treatment, focused on lifestyle changes; these effects were evaluated 12 months’ post baseline. The parent-group treatment was significantly more effective for reduction in Body Mass Index (BMI) z-scores (primary outcome) compared to the standard treatment [12]. Similar to Hayes et al.’s trial [8], this intervention did not specifically target picky eating, but secondary outcomes of the trial encompassed changes in child eating behaviors, including picky eating. Considering the potential role of the parent-child relationship in picky eating, the present analysis evaluates whether the parent-group treatment led to reductions in children’s picky eating behaviors, and whether reductions in picky eating facilitated weight reduction.

Picky eating during childhood has been the focus of several research studies in recent years, with most studies assessing picky eating through questionnaires that measure parents’ perceptions of their child’s actions. However, no gold-standard measurement of picky eating exists. As reviewed by Taylor et al. [1] a variety of subscales and questions are used in research on picky eating, leading to inconsistencies in research findings, most notably in prevalence studies. Despite these inconsistencies, to date, no study has compared how different picky eating questionnaires compare when completed by the same population. Thus, in the present study, we evaluated picky eating in an obesity intervention for preschool-aged children with two parent-reported instruments: The FF subscale from the CEBQ and a subset of 5 items from the Lifestyle Behavior Checklist (LBC) [13, 14]. The FF-scale in the CEBQ is more commonly used and has been validated for the purpose of assessing picky eating [1, 15]. Although both subscales have been used to assess picky eating in preschoolers in previous studies, they are constructed somewhat differently. In the CEBQ parents are asked to describe their child’s eating behavior by evaluating six statements (e.g. my child enjoys a wide variety of foods), while in the LBC parents instead respond to the statement “To what degree has this behavior in your child been a problem for you?” (e.g. refuses to eat certain food). Given that the LBC emphasizes the parents’ own perception of the child’s behavior being a problem, mothers and fathers responded to this questionnaire separately. This provides us with the opportunity to study potential gender differences.

### Aim and hypotheses

The aim of this analysis is to evaluate the role of picky eating in a randomized controlled obesity intervention for preschoolers using subscales from two instruments: The CEBQ and the LBC.

We hypothesize that picky eating will decrease after the intervention, particularly in the parent-group treatment. Further, we hypothesize that a decrease in picky eating will be associated with changes in child weight status. We also hypothesize that baseline levels of picky eating, in this treatment-seeking sample of children with obesity, will be associated with lower baseline BMI z-scores and lower intake of food items such as vegetables. We further hypothesize that baseline levels of picky eating will be associated with changes in child weight status over treatment.

With regards to the two measurements of picky eating, we hypothesize that they will be correlated and generate similar results. We further hypothesize that results for picky eating measured separately for mothers and fathers with the LBC will render similar results with regards to perceived levels at baseline and changes during treatment.

## Methods

### Intervention study design

The More and Less (ML) study is a parallel open labeled RCT aiming to evaluate the effectiveness of a group program for parents of preschoolers with obesity, focused on evidence-based parenting practices, compared to standard treatment focused on lifestyle changes. The methodology and study design have been described in detail in previous publications [12, 16]. Families (n = 177) with preschool-aged children diagnosed with obesity were randomized either to the parent-group treatment (with or without follow-up sessions) or to the standard treatment.

The parent-group treatment was developed to enhance evidence-based parenting practices among parents of preschool-aged children with obesity. The treatment was delivered by dieticians in 10 weekly 90-min sessions. Children did not participate in the groups. Each of the weekly sessions focused on a specific parenting practice to support a healthy home environment, along with a healthy lifestyle component (e.g. healthy eating, physical activity), which were presented by group leaders, discussed by parents, and practiced through role playing. Examples of parenting practices included in the program are encouragement, monitoring, positive involvement, limit setting and problem solving strategies along with regulation of emotional expression [16]. After the weekly sessions concluded, a randomly selected subgroup of parents (n = 44) received follow-up booster phone calls every 4–6 weeks. The booster phone calls provided parents with encouragement and reminders of the parenting techniques practiced in the group sessions. Findings specific to the booster group are not analyzed in the present study.

The standard treatment was the one currently offered to children with obesity in Stockholm, Sweden and is based on the action plan for childhood obesity in Stockholm County [17], focusing on establishing healthy food choices and active lifestyle habits. In the RCT, the standard treatment was delivered to families (parents and child together) by pediatricians (the first visit) and pediatric nurses (follow-up visits) in outpatient pediatric clinics. The families were offered at least 4 visits over 12 months.

### Participants

Families were recruited between March 2012 and March 2016, data were collected at 4 time-points: at baseline and at 3, 6 and 12 months. Participant recruitment was conducted mainly through primary child health care and outpatient pediatric clinics in Stockholm County, Sweden. The study included children who were 4 to 6 years old, who were diagnosed with obesity according to age- and sex-specific international cut-offs [18], and who were not diagnosed with any chronic disease or developmental problem. In addition, to be included in the study, parents needed to be sufficiently proficient in Swedish to fill out questionnaires and participate in a discussion-centered treatment [16].

### Measurements and outcomes

#### Two parent reported measurements of picky eating

The study used two measurements that have been used to evaluate picky eating in children in previous studies: the more established and commonly used FF subscale from the CEBQ and the less commonly used five items from the LBC [1]. Mothers and fathers completed the LBC separately and one parent per family completed the CEBQ (in 80% of cases, mothers completed the CEBQ). Overall, to reduce participant burden in the RCT, one parent per family filled out questionnaires focusing on the child; however, questionnaires focusing on the parents were filled out separately by both parents.

The CEBQ has been widely used to assess eating behaviors among preschoolers [13] and was validated in Sweden for this age group [19]. Among the eight subscales included in the CEBQ, the six-item FF subscale focuses on food neophobia and picky eating. In the present study, the Cronbach’s alpha values of the scale at baseline, and at 3, 6 and 12 months were 0.86, 0.86, 0.90 and 0.87. The FF subscale in the CEBQ consist of 6 statements evaluated on a five-point Likert scale, from “never” to “always”. Parents are asked to describe their child’s eating behavior.

- My child refuses new food at first
- My child enjoys tasting new foods (Reversed scale)
- My child enjoys a wide variety of foods (Reversed scale)
- My child is interested in tasting food s/he hasn’t tasted before (Reversed scale)
- My child decides that s/he doesn’t like food, even without tasting it
- My child is difficult to please with meals

The LBC assesses parents’ perceptions of children’s obesity-related problem behaviors [14] and has been validated in Sweden for the preschool age group [19]. Of the questionnaire’s 25 items, five have been used to assess picky eating in previous research [20]. Mothers and fathers were asked to respond separately to the statement: “To what degree has this behavior in your child been a problem for you” on a 7-point scale, from “not at all” to “very much”. The Cronbach’s alpha values for the mothers’ responses at baseline, and at 3, 6, and 12 months were 0.76, 0.80, 0.70 and 0.75. For the fathers’ responses, the Cronbach’s alpha values were 0.82, 0.83, 0.73 and 0.84, respectively. The five items are:

- Whinges or whines about food
- Yells about food
- Throws a tantrum about food
- Refuses to eat certain foods (i.e. fussy eating)
- Argues about food (e.g. when you say no more)

#### Child BMI z-scores

Child height was measured by trained health care professionals to the nearest 0.1 cm using a fixed stadiometer. Children were weighed to the nearest 0.1 kg wearing underwear. BMI was calculated based on weight and height. Child BMI z-score was derived from age and sex specific reference data [18].

#### Child food frequency questionnaire

A short version of a parent-reported food frequency questionnaire (FFQ) was also included (only baseline data analyzed in the present study). A more extensive version of the FFQ was included in the Swedish National Food Agency’s national dietary survey targeting children aged 4, 8 and 11 [21]; a similar FFQ was also previously used and validated against 4-day food records in a primary prevention trial of childhood obesity in Swedish child health centers [22]. This short version assessed children’s consumption frequency of 10 food items (fresh fruits, vegetables, pizza/hamburger, fish, ice cream, cakes/buns/cookies, soft drinks, juice, sweets, chips/snacks), with 13 response categories ranging from once per month or less to four times per day or more. Frequency equivalents were calculated for daily, weekly and monthly consumption.

#### Socio-demographics

Parental educational and income level, non-Swedish origin (parent and both grandparents born in a country other than Sweden, or parent born in Sweden and grandparents born abroad) and parental weight and height (BMI) were self-reported at baseline.

### Statistical methods

To evaluate the association between picky eating measured with the two subscales, data were first matched to ensure that the same parent had completed both the LBC and the CEBQ (six children lacked LBC data). In 77% of the cases this was the mother, in 20% the father and in 3% both parents (in these cases, the mothers’ LBC data were selected for analysis). Then, a mixed effects model was used, with the LBC as the dependent variable and the CEBQ as the only covariate. It also included a fixed intercept, a fixed coefficient for CEBQ, a child-specific random intercept, and a child-specific random coefficient for CEBQ. We assumed the random intercept and random time coefficient followed a bivariate normal distribution with an unstructured two-by-two covariance matrix.

Means (SD) across each food frequency item were calculated. The pair-wise Pearson’s correlation coefficient was used to study the associations between baseline picky eating (measured with the CEBQ and the LBC respectively) and baseline BMI z-scores as well as the reported frequency of intake of specific food items.

Linear mixed-effects models were used to assess if picky eating behaviors changed over time and between the two treatment groups, if changes in picky eating were associated with changes in BMI z-scores over time, and if baseline picky eating had an effect on BMI z-scores in the two treatment groups over time. These models permitted assessing within- and between-children variability, while taking into account the within-child temporal dependence in the outcome variables. Separate models were used for the different outcome variables considered. All the models contained a fixed intercept, a fixed coefficient for study time, a fixed coefficient for the binary indicator of the treatment group, and a fixed coefficient for the interaction between the time coefficient and the treatment indicator. Study time entered the model as a numeric variable. The models also included a child-specific random intercept and a child-specific random coefficient for study time. We assumed the random intercept and random time coefficient followed a bivariate normal distribution with an unstructured two-by-two covariance matrix. The statistical analyses were performed with Stata version 15 (StataCorp, College Station, TX, USA).

## Results

### Sample characteristics

Three out of the 177 enrolled children were excluded post randomization due to receiving a diagnosis that affected the child’s physical development. In total, 37 (21%) of the children were excluded post-intervention because they did not attend a follow-up assessment. Baseline data from the CEBQ FF subscale were available for 130 children, see Table 1 for the background characteristics. The mean age of the children was 5.2 years (SD 0.7, range 4–6 years) and 54% were girls. A majority (80%) lived with both parents. More than half of the parents were of non-Swedish background and about 40% had a university degree. The majority of parents had a monthly income lower than the mean in Stockholm County, Sweden, according to official statistics. Picky eating data from the LBC questionnaire were available from 124 mothers and 107 fathers at baseline.

**Table 1:** Characteristics of study sample at baseline

	Total sample (n = 130)	Parent-group treatment (n = 65)	Standard treatment (n = 65)
	% or mean (SD)		
Child			
Age (years)	5.2 (0.7)	5.2 (0.8)	5.3 (0.7)
Sex (girl)	54	42	66
Living with both parents	80	82	79
BMI z-score	2.9 (0.6)	3.0 (0.7)	2.9 (0.6)
Mother			
Age (years)	36.5 (5.5)	37.0 (5.4)	36.0 (5.7)
BMI	28.2 (5.8)	28.7 (6.2)	27.6 (5.3)
Foreign origin	59	59	58
University degree	43	44	42
Income (SEK)			
< 20,000	62	54	70
20,000 < 30,000	29	33	25
30,000 < 40,000	7	11	3
> 40,000	2	2	2
Father			
Age (years)	39.8 (7.3)	40.7 (8.0)	38.9 (6.5)
BMI	29.6 (4.4)	29.7 (4.5)	29.5 (4.4)
Foreign origin	56	58	54
University degree	40	40	41
Income (SEK)			
< 20,000	36	37	34
20,000 < 30,000	46	42	50
30,000 < 40,000	13	12	14
> 40,000	5	9	2

Missing values: mother BMI = 4, Father BMI =14, Mother age = 3, father age = 15, mother university degree = 3, father university degree = 13, mother foreign background = 2, father foreign background = 12, mother income = 3, father income = 15. Foreign origin, parent and both grandparents born in a country other than Sweden, or parent born in Sweden and grandparents born abroad. Abbreviations: BMI, Body Mass Index. SEK, Swedish kronor. Income, in 2015, the mean monthly income level in Stockholm County, Sweden was 33,600 SEK (men) and 26,400 SEK (women)

### Change in picky eating across treatments

Table 2 shows mean values for picky eating measured with the CEBQ and the LBC at baseline, and at 3, 6, and 12 months post baseline. Mixed effects modeling showed that picky eating measured with the CEBQ did not change over time and no differences were found in the time trend between the parent-group treatment and the standard treatment. Similarly, picky eating measured with the LBC did not change over time in any of the treatment groups. At baseline, the mothers’ LBC questionnaires yielded significantly higher picky eating scores compared to the fathers’ questionnaires (p = 0.019), but no differences were found in change over time between the mothers’ and fathers’ questionnaires.

**Table 2:** Mean values for picky eating measured with the CEBQ^1^ and the LBC^2^ at baseline and post treatment

	Baseline			3-months			6-months			12-months		
	n	mean	SD	n	mean	SD	n	mean	SD	n	mean	SD
CEBQ FF subscale^1^												
Whole sample	130	2.6	0.8	92	2.6	0.7	101	2.6	0.8	103	2.6	0.8
Parent-group treatment	65	2.6	0.8	46	2.5	0.8	52	2.6	0.8	54	2.6	0.8
Standard treatment	65	2.5	0.7	46	2.7	0.7	49	2.6	0.8	49	2.5	0.7
LBC Mother^2^												
Whole sample	124	2.8	1.3	92	2.5	1.3	99	2.4	1.0	97	2.4	1.1
Parent-group treatment	63	2.8	1.3	46	2.3	1.1	50	2.4	1.1	50	2.3	1.2
Standard treatment	61	2.7	1.4	45	2.7	1.3	49	2.5	1.0	47	2.4	1.0
LBC Father^2^												
Whole sample	107	2.5	1.3	83	2.5	1.3	90	2.2	1.0	83	2.3	1.2
Parent-group treatment	55	2.5	1.2	41	2.5	1.2	44	2.3	0.9	43	2.3	1.1
Standard treatment	52	2.4	1.3	42	2.4	1.3	46	2.1	1.0	40	2.2	1.2

^1^ CEBQ: Child Eating Behavior Questionnaire, picky eating measured on a scale from 1 to 5 on the Food Fussiness subscale (6 items)

^2^ LBC: Lifestyle Behavior Checklist, picky eating measured on a scale from 1 to 7 (5 items)

### Role of picky eating across treatments

**Fig. 1:**
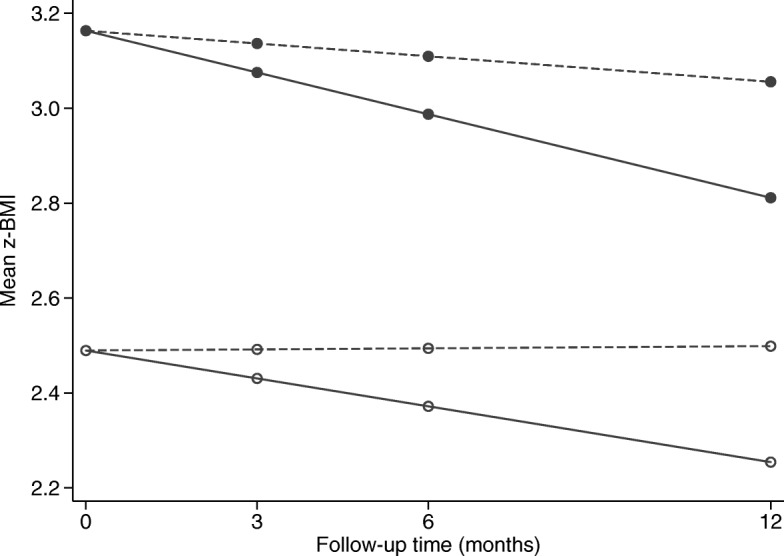
Estimated change in mean BMI z-scores over the follow-up time in the parent-group treatment (solid lines) and in the standard treatment (dashed line) in two individuals: one with low level of baseline picky eating, CEBQ equal to 1 (solid dots) and the other with high level of baseline picky eating, CEBQ equal to 5 (hollow dots)

Changes in picky eating measured with the CEBQ or the LBC showed no associations over time with changes in BMI z-scores. For picky eating measured with the LBC, baseline levels of picky eating had no effect on changes in BMI z-scores. However, an effect was detected for picky eating measured with the CEBQ (Fig. 1). While BMI z-scores significantly declined in the parent-group treatment overall, the decline in weight was significantly steeper in children with a lower degree of baseline picky eating (slope coefficient: − 0.03, p = 0.001) compared to children with a higher degree of picky eating (slope coefficient: − 0.02, p = 0.087). In the standard treatment group, changes in BMI z-scores were minor and independent of the degree of baseline picky eating (slope coefficient among children with a lower degree of baseline picky eating: − 0.01, p = 0.280; among children with a higher degree of baseline picky eating: 0.01, p = 0.948).

### Picky eating measured with subscales from the CEBQ and the LBC

**Fig. 2:**
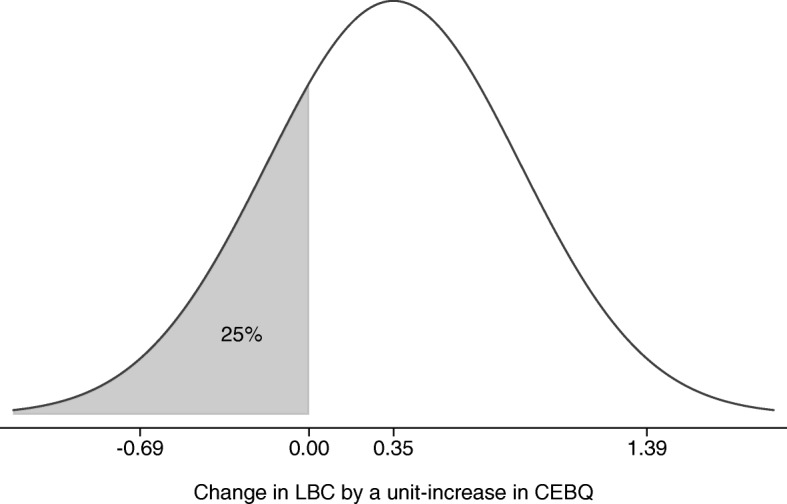
Model-implied distribution across individuals of the change in LBC by increased in CEBQ

In the total sample, over all measured time points, picky eating measured with the FF subscale of the CEBQ showed a moderate significant positive correlation with picky eating measured with the 5 items from the LBC (coef. 0.35, p = < 0.001, SD 0.52); however, the slope varied highly between individuals. This is illustrated in Fig. 2 which also shows that the probability of a negative association between the two measurements was 25%. For these children, the degree of picky eating measured with the CEBQ diverged in the opposite direction from the degree of picky eating measured with the LBC.

At baseline, picky eating measured with the CEBQ was negatively associated with child BMI z-scores and intake of vegetables. No significant associations were found for picky eating measured with the LBC, regardless of whether fathers or mothers completed the questionnaire (see Table 3).

**Table 3:** Mean scores (SD) and correlations between the CEBQ^1^, the LBC^2^, BMI z-scores and food intake at baseline

		Correlations with picky eating		
	Mean (SD)	CEBQ FF-subscale^1^	LBC^2^ Mothers	LBC^2^ Fathers
Child BMI z-score	2.9 (0.6)	−0.211*	−0.013	−0.099
Food frequency				
Fresh fruits (per day)	1.7 (0.9)	−0.117	− 0.026	− 0.115
Vegetables (per day)	1.6 (1.0)	−0.371**	− 0.175	− 0.061
Pizza/hamburger (per month)	2.2 (1.5)	0.110	0.102	0.184
Fish (per week)	1.9 (2.1)	−0.169	− 0.018	− 0.104
Ice cream (per month)	4.1 (4.5)	0.143	0.002	0.048
Cakes/buns (per week)	1.2 (1.1)	0.074	−0.059	0.061
Soft drinks (per week)	1.4 (2.2)	0.119	0.029	0.150
Juice (per week)	2.1 (3.4)	0.044	−0.016	0.173
Sweets (per week)	1.2 (0.9)	0.069	−0.131	0.056
Chips/snacks (per week)	0.9 (0.8)	0.027	0.047	0.102

*P < 0.05, **P < 0.01

^1^ CEBQ: Child Eating Behavior Questionnaire, picky eating measured on a scale from 1 to 5 on the Food Fussiness subscale (6 items)

^2^ LBC: Lifestyle Behavior Checklist, picky eating measured on a scale from 1 to 7 (5 items)

## Discussion

The aim of this analysis was to evaluate the role of picky eating in a randomized controlled obesity intervention for preschoolers using subscales from the CEBQ and the LBC. Whether measured with the CEBQ or the LBC, picky eating did not change during a randomized controlled obesity intervention among preschoolers, and no associations were found between changes in picky eating and changes in BMI z-scores. While the parent-group treatment led to significantly greater reductions in children’s BMI z-scores compared to the standard treatment [12], children with a higher degree of picky eating at baseline, measured with the CEBQ, experienced smaller reductions in weight. Further, children with a higher degree of picky eating at baseline, measured with the CEBQ, had a lower BMI z-score and consumed less vegetables. No significant associations were found for picky eating measured with the LBC. Our results also show that measuring picky eating with the CEBQ and the LBC generated different results, and that the gender of the parent who completes the questionnaire may affect the results.

A bi-directional relationship between parents’ and children’s behaviors in relation to picky eating has been proposed recently [11]. Thus, we hypothesized that the parent-group treatment, focusing on parenting practices, would be more effective in reducing picky eating behaviors, and that this reduction would be associated with the greater weight loss seen in this group. In the parent-group treatment, parents were encouraged to work with behaviors they found challenging. Strategies to overcome picky eating-related behaviors, such as convincing children to eat vegetables, were a common concern. Parents were taught different techniques, such as how motivational charts can be used to encourage the child to taste and eat different foods. In contrast to our hypothesis, we did not see any change in picky eating over time for any of the treatment groups. This finding also contrasts with the findings of Hayes et al. [8], who found a significant reduction in CEBQ-measured picky eating post treatment, and an effect of reduction in picky eating on greater weight loss in the follow-up. This contrast may due to differences in study design: the US study included children with overweight as well as obesity, and our study had a longer follow-up period of one year, compared to immediately post treatment. Perhaps most importantly, there was an age difference between the participating children. The mean age of the children in the study by Hayes et al. was 9.4 years (SD 1.2) while the children in our study had a mean age of 5.2 (SD 0.7). The trajectories of picky eating in children are not fully understood. The behavior has been described as part of normal development in preschool-aged children but a substantial group of persistent picky eaters continues to have problems beyond the preschool age [10]. Thus, compared to 5-year-olds for whom picky eating may be part of a developmental process, small changes in picky eating scores among 9-year-olds may have a greater effect on the outcomes of obesity interventions.

According to our hypothesis and in contrast to Hayes et al. [8], we found that baseline picky eating, measured with the CEBQ FF-subscale, had an effect on changes in BMI z-scores (no effect for baseline LBC picky eating). As previously described, the parent-group treatment was significantly more effective in reducing BMI z-scores 12 months post baseline as compared to the standard treatment [12]. However, the parent-group treatment was significantly less effective for children with higher baseline picky eating measured with the FF subscale of the CEBQ. This indicates that degree of baseline picky eating might be an extra barrier for treatment success among preschoolers in weight reduction programs. It also underscores that clinicians should consider different behavioral phenotypes pre-treatment and possibly adapt treatment accordingly [23]. Of note, the standard treatment was ineffective independent of the degree of baseline picky eating measured with the FF subscale of the CEBQ.

The present study found, in line with our hypothesis, that higher levels of picky eating (measured with the CEBQ FF subscale) were associated with a lower intake of vegetables, but no other significant associations emerged. In our previous study, we found that preschoolers with overweight or obesity who were classified as picky eaters had lower degrees of obesogenic eating behaviors (e.g., lower enjoyment of food and lower food responsiveness) compared to preschoolers with overweight or obesity who were not classified as picky eaters [6]. Importantly, differences between the groups extended to other lifestyle behaviors, as children with a higher degree of picky eating also complained more about being physically active and had more problematic screen time behaviors [6]. Although all participating children were diagnosed with obesity, the present study found that a higher baseline degree of picky eating was associated with a lower baseline BMI z-score; this may be related to this group’s less steep weight reduction slope at follow-up.

Picky eating is a common concern among parents of preschool-aged children independent of weight status [6]. The present study showed that picky eating plays a role in the outcomes of obesity treatment for preschoolers. While the factors underlying the relationship between picky eating and obesity intervention outcomes require further investigation, it is possible that, for preschool-aged children with obesity and picky eating, behavioral changes may be more difficult to initiate and sustain, leading to a lower reduction in weight status. To formulate evidence-based treatment adaptations that respond to the needs of children with obesity and picky eating, future research should examine how picky eating influences families’ capacity to engage with the various components of childhood obesity treatment. Notably, a recent study has shown that framing picky eating as a key issue of concern may increase parents’ motivation to participate in preventive interventions [24]. This could also be true for parent-based obesity interventions, and additional studies should evaluate how addressing picky eating in childhood obesity prevention and treatment might affect parents’ engagement and treatment success.

From a methodological perspective, our results confirm that different measurements of picky eating may lead to inconsistencies in research findings [1, 2]. Although, as hypothesized, a significant correlation was found between the CEBQ and the LBC measurements of picky eating, further examinations revealed a high variability between subjects. This means that higher CEBQ values were not always correlated with higher LBC values. Thus, a child reported as a picky eater on one scale would not be reported as a picky eater using the other scale. This finding underscores that research studies on picky eating should clearly state which measurement has been used and why. Moreover, this finding suggests that it is essential to develop a standardized definition of picky eating and measurements that can facilitate comparisons between different research studies. Overall, significant results were shown using the more established FF subscale from the CEBQ to measure picky eating compared to the subset of items from the LBC. This indicates the benefit of choosing a measurement that has been developed and validated for this purpose and is more commonly used [1, 7, 15]. Although a previous study has found negative associations between LBC-measured picky eating and fruit and vegetable intake [20], in the present study, at baseline, only picky eating measured with the FF subscale of the CEBQ showed associations with lower BMI z-scores and a lower intake of vegetables. This finding supports the validity of the CEBQ FF subscale, because picky eating has been associated in previous research with lower weight in children as well as a lower intake of vegetables [1–3].

While the two subscales might seem similar at first glance, the LBC focuses on parents’ perceptions of children’s food-related emotional expressions, such as yelling, arguing and throwing tantrums, whereas the CEBQ focuses on children’s behaviors in relation to food, such as refusing, enjoying and deciding. Most measurements of picky eating are parent-reported and, in addition to the influence that choice of measurement instrument may have on outcomes, our study showed that the gender of the parent who completes the questionnaire may also affect the results – a finding that contrasts with our hypothesis. At baseline, mothers’ LBC questionnaire yielded significantly higher picky eating scores compared to fathers’ questionnaires. Although the paternal role increasingly includes feeding and caring for children [25, 26], fathers are still underrepresented in pediatric obesity research [27]. While the present study contributes a very small piece to this puzzle, to our knowledge, no previous study has compared mothers’ and fathers’ perceptions of children’s picky eating behaviors.

A particular strength of the study was its randomized controlled design, as well as its longitudinal design, which included the collection of follow-up data at 12 months post baseline. Another strength was the study’s use of the CEBQ and the LBC to measure picky eating, which allowed for a comparison of these measures and for more robust analysis. The study’s main limitation was the variation in the number of missing values at different time-points – a limitation linked to the study’s longitudinal design. Power for the RCT was calculated for the difference in primary outcome (BMI z-scores); therefore, there is a risk that the analysis did not have enough power to capture changes in picky eating.

## Conclusion

This is the first study to examine picky eating and its relation to weight change in preschoolers after obesity treatment. Analyzing data from a randomized controlled obesity intervention, where parent-group treatment was compared to standard treatment, our findings show that neither the standard treatment nor the parent-group treatment reduced the degree of picky eating in preschoolers with obesity. Notably, our findings show that picky eating at baseline may affect obesity intervention outcomes. While the parent-group treatment led to significantly greater reductions in weight overall, children with a higher degree of picky eating at baseline displayed a lower degree of weight loss. These results suggest that, prior to commencing childhood obesity treatment, clinicians should consider differences in children’s behavioral phenotypes, such as picky eating, and adapt treatment plans according to these children’s needs. Our findings also show that, from a methodological point of view, it is crucial to consider which questionnaire is used to measure picky eating: for one-fourth of the children, CEBQ scores and LBC scores indicated diverging results. Additionally, our findings suggest that selecting either mothers or fathers to complete the CEBQ or LBC picky eating subscales may affect the results.

## Data Availability

These data are not publically available, but investigators that wish to access specific parts of the data should contact the corresponding author.

## Abbreviations

BMI: Body Mass Index
CEBQ: Child Eating Behavior Questionnaire
FF: Food Fussiness
FFQ: Food Frequency Questionnaire
LBC: Lifestyle Behavior Checklist
ML: The More and Less study
RCT: Randomized Controlled Trial
SD: Standard deviation
